# The role of health literacy in intervention studies targeting children living with overweight or obesity and their parents—a systematic mixed methods review

**DOI:** 10.3389/fped.2024.1507379

**Published:** 2025-01-22

**Authors:** Heidi Holmen, Tone Nygaard Flølo, Christine Tørris, Astrid Torbjørnsen, Kari Almendingen, Kirsti Riiser

**Affiliations:** ^1^ Department of Nursing and Health Promotion, Faculty of Health Sciences, Oslo Metropolitan University, Oslo, Norway; ^2^ Intervention Centre, Oslo University Hospital, Oslo, Norway; ^3^ Department of Surgery, Voss Hospital, Bergen Health Trust, Bergen, Norway; ^4^ Department of Rehabilitation Science and Health Technology, Faculty of Health Sciences, Oslo Metropolitan University, Oslo, Norway; ^5^ Department of Child and Adolescent Health Promotion Services, Norwegian Institute of Public Health, Levanger, Norway

**Keywords:** review, health literacy, interventions, childhood obesity, weight management

## Abstract

**Background:**

Excess weight and obesity are increasing among children. Health literacy has been suggested as a feasible concept for enabling informed health choices in weight management interventions for children and their parents. Knowledge of the skills necessary for a child to maintain new health behaviors is limited and the role of health literacy remains unclear. Thus, there is a need to summarize the effects of and experiences with interventions that include health literacy components to guide the development of effective, future weight-related interventions.

**Aim:**

This review aims to identify how health literacy is integrated into studies of interventions targeting children with excess weight or obesity and/or their parents and to appraise the identified literature.

**Methods:**

We conducted a systematic mixed methods review, with searches in Medline, CINAHL, Cochrane, EMBASE, ERIC, PsycINFO, and Web of Science. We included studies of interventions published after 2013 that targeted children under 19 years with excess weight or obesity and/or their parents, where health literacy played a role. Results from the included studies were integrated using qualitative data transformation techniques, followed by a narrative summary.

**Results:**

We identified 7,910 citations. Four reports met our inclusion criteria and were included for review. These reports included a total of 402 children. Health literacy was assessed at baseline in two studies and measured as an outcome over time in one study. Methodological quality varied among the retained reports, with differences observed in study design, risk of bias and data collection methods. The reports highlight the need to adapt weight management treatments to the individual level of health literacy in children and their families to first ensure active participation in their treatment and second ensure long-term compliance with necessary lifestyle-related changes.

**Discussion:**

Surprisingly, little attention has been paid to the importance of health literacy in weight management programs targeting children and their families. Seemingly, treatments tailored to the individual level of health literacy have not been prioritized in research. Addressing health literacy in children's weight management continues to be a multifaceted and ambitious mission. Future research should focus on integrating health literacy into weight management interventions in a systematic and theory-driven manner, ensuring that these interventions are tailored to the specific needs of children and their families and can sustain behavior change over time.

**Systematic Review Registration:**

https://www.crd.york.ac.uk/prospero/display_record.php?RecordID=478957, identifier: CRD42023478957.

## Background

Obesity is a major threat to public health worldwide. In recent decades, the prevalence of both overweight and obesity has increased in all age groups (1). Of particular concern is the increase among young people; overweight and obesity are currently affecting one in three European children (2). In some high-income countries, the rising trend has flattened out (3). However, this trend is restricted to children living in families with a high socioeconomic status (4). Childhood overweight and obesity are often associated with a range of negative physical and psychosocial health effects (5), including impaired quality of life and a positive association with the onset of other noncommunicable diseases, such as diabetes, cardiovascular diseases, several cancers, and perpetuated obesity in adulthood (6–9).

A child needs adequate nutritional care starting from prenatal age (10). Growing up, the primary causes of overweight and obesity are an unbalanced diet and a lack of physical activity. Children tend to inherit similar lifestyle-related habits as their parents. Therefore, a child with excess weight or obesity is likely to have at least one parent with the same condition (11). Since obesity is still on the rise (1), there is a need for new and sustainable weight management interventions to support children with excess weight or obesity and their families (12, 13).

Suggestions have been made that interventions targeting lifestyle-related conditions, such as obesity, should be developed at the family level and as early in childhood as possible (11). The core of obesity treatment for children lies with lifestyle interventions (14), which frequently includes educational elements intended to encourage a healthy lifestyle by enhancing participants' knowledge about health, physical activity, and nutrition (15–17). The vast amount of information, educational, and theoretical resources regarding weight-related behavior and weight management can be overwhelming, particularly for children and families, who may struggle to understand and navigate this information effectively and make meaning of it in their own lives (18). However, few studies have explored the health literacy skills necessary for a child and family to act on this knowledge during and after an intervention. This might partly explain why maintaining such a lifestyle change is challenging.

Health literacy refers to the personal characteristics and social resources needed for individuals and communities to access, understand, appraise, and use information and services to make decisions about health, including the capacity to communicate, assert, and enact those decisions (19). Research has shown that both a child's and a parent's health literacy can significantly impact weight management efforts. Lower health literacy in children has been associated with higher body mass index (BMI) and greater likelihood of overweight or obesity (20, 21). Similarly, parents with lower health literacy may struggle to comprehend health information which can lead to poor dietary choices, impaired health and higher rates of excess weight or obesity in their children (22). Therefore, health literacy plays a crucial role in empowering both children and their parents to make informed health decisions and effectively manage weight (21, 23, 24). By broadening their skills beyond acquiring information, children and parents may be able to act in the best interests of their health, emphasizing the importance of decision-making, communication, and the skills needed to navigate health information and health services (22, 25–27).

The interplay between health literacy and socioeconomic status also significantly affects the risk of excess weight and obesity in children (22). Health literacy constitutes a significant social health determinant, with lower health literacy often found in groups with lower socioeconomic status. These groups are also at a higher risk of excess weight and obesity, highlighting a social gradient in childhood obesity (28–30).

Despite evidence on the associations between health literacy and excess weight in children and their parents, there is limited research on the effect of health literacy interventions on obesity management (23). Previous research suggests health literacy as a feasible concept for weight-related interventions to enable children to make informed health choices (31, 32). However, knowledge regarding interventions that support health literacy in children with excess weight and obesity and their parents remains unclear and has not been systematically reviewed. To address this gap, we conducted a systematic mixed methods review to investigate the role of health literacy in studies of interventions for children with excess weight or obesity and clarify the characteristics, content, and outcomes in these studies.

## Objective

This review aims to identify how health literacy is integrated into studies of interventions targeting children with excess weight or obesity and/or their parents and appraise the identified literature.

## Methods

### Design

Following the Joanna Briggs Institute guidelines, we conducted a systematic review using a mixed methods convergent design (33). Studies were included irrespective of design, and the results of the retained studies were integrated using qualitative data transformation techniques (34). A protocol was published in the International Prospective Register of Systematic Reviews (PROSPERO) on November 14th, 2023 [CRD42023478957]. The Preferred Reporting Items for Systematic Reviews and Meta-Analyzes (PRISMA) statement guided our reporting (35) (Supplementary File S1).

### Eligibility criteria

Eligibility criteria were set using the Population, Concept, Context (PCC) tool (36) (Table 1), targeting primary reports of scientific research in which health literacy was integrated as a predictor, a means, or an outcome in studies of interventions targeting children with excess weight or obesity and/or their parents. If reports included the same study population and intervention, we prioritized including the report that provided the most comprehensive and detailed information relevant to our research question. Qualitative and quantitative studies were eligible for inclusion. To ensure the relevance of the identified literature, we searched for literature published from January 1st, 2013, until October 25th, 2023. The search string targeting population was tailored from a search string for an umbrella review conducted by the research group (blinded), consisting of some similar elements, and the search string for context was adapted from one used in a similar health literacy systematic review (30). A complete overview of all searches is provided in the Supplementary Material S2.

**Table 1:** Inclusion criteria: population, concept and context (PCC) (36).

	Inclusion criteria
Population (P)	Children under the age of 19 years with excess weight or obesity, according to Cole et al. (37), and/or their parents.
Concept (C)	The role of health literacy, understood according to the definition of Dodson et al. (19), comprises the personal characteristics and social resources needed for individuals and communities to access, understand, appraise, and use information and services to make decisions about health, including the capacity to communicate, assert and enact these decisions.
Context (C)	Intervention studies for children with excess weight or obesity and/or their parents.
Design	Original research applying qualitative, quantitative, mixed- or multi-methods designs.
Language	English, Scandinavian.

### Information sources

A systematic search was conducted in Medline (Ovid), CINAHL (EBSCOhost), Cochrane (limited to Trials), EMBASE (Ovid), ERIC (EBSCOhost), PsycINFO (Ovid), and Web of Science (Core collection). The final search, depicted in Supplementary File S2, was conducted by a university librarian and peer-reviewed according to the Peer Review of Electronic Search Strategies (PRESS) guidelines (38).

### Data selection

The search results were exported to the citation and reference management tool EndNote for automatic deduplication, followed by manual control. The remaining citations were imported to software for systematic review management, Covidence, for independent, blinded screening in pairs (39). Titles and abstracts were screened in randomly assigned pairs to assess eligibility, and nonexcluded citations were uploaded for a full-text assessment by two independent reviewers. For publications with discordant results or other uncertainties related to eligibility criteria, an additional reviewer performed an independent assessment with consecutive discussions to reach a consensus. The process was recorded in a PRISMA flow chart (35).

### Methodological appraisal of individual sources of evidence

Methodological quality was appraised using the relevant checklist available through the Mixed Methods Appraisal Tool (40). This tool contains two initial screening questions, similar for all designs, followed by design-specific versions for qualitative, quantitative nonrandomized, quantitative descriptive, mixed-method designs, or quantitative randomized controlled trials. All criteria are rated as either “yes”, “no”, or “can't tell”. Methodological appraisal was performed by HH and controlled by TNF. Utilizing the Grading of Recommendations Assessment, Development, and Evaluation (GRADE) tool (41) was not deemed relevant for this study, as it primarily assesses the quality of evidence in systematic reviews of interventions. In contrast, our study focused on a mixed methods review, incorporating both qualitative and quantitative studies.

### Data charting

Consistent with this study's aim, the research team developed a data extraction template. The template contained the authors, year of publication, country of origin, aim of the study, design and methods, study population and sample size, theoretical framework for the health literacy intervention, details of the health literacy intervention, and findings related to the research questions of our review. Data were extracted by HH and checked by TNF to ensure reliability before a joint discussion regarding any discrepancies.

### Synthesis of results

The results are presented descriptively in text and tables. Data from the results section of the reports were extracted by HH. The results were transformed into a qualitative text format before synthesis (34, 40). The data were analyzed consistent with the steps of the thematic synthesis (34, 42). To understand the material, the first (HH) and last authors (KR) read the extracted data several times. The data were then synthesized based on the findings' relevance to the study aim and the role of health literacy in the included reports.

## Results

### Overview of the search results

**Figure 1:**
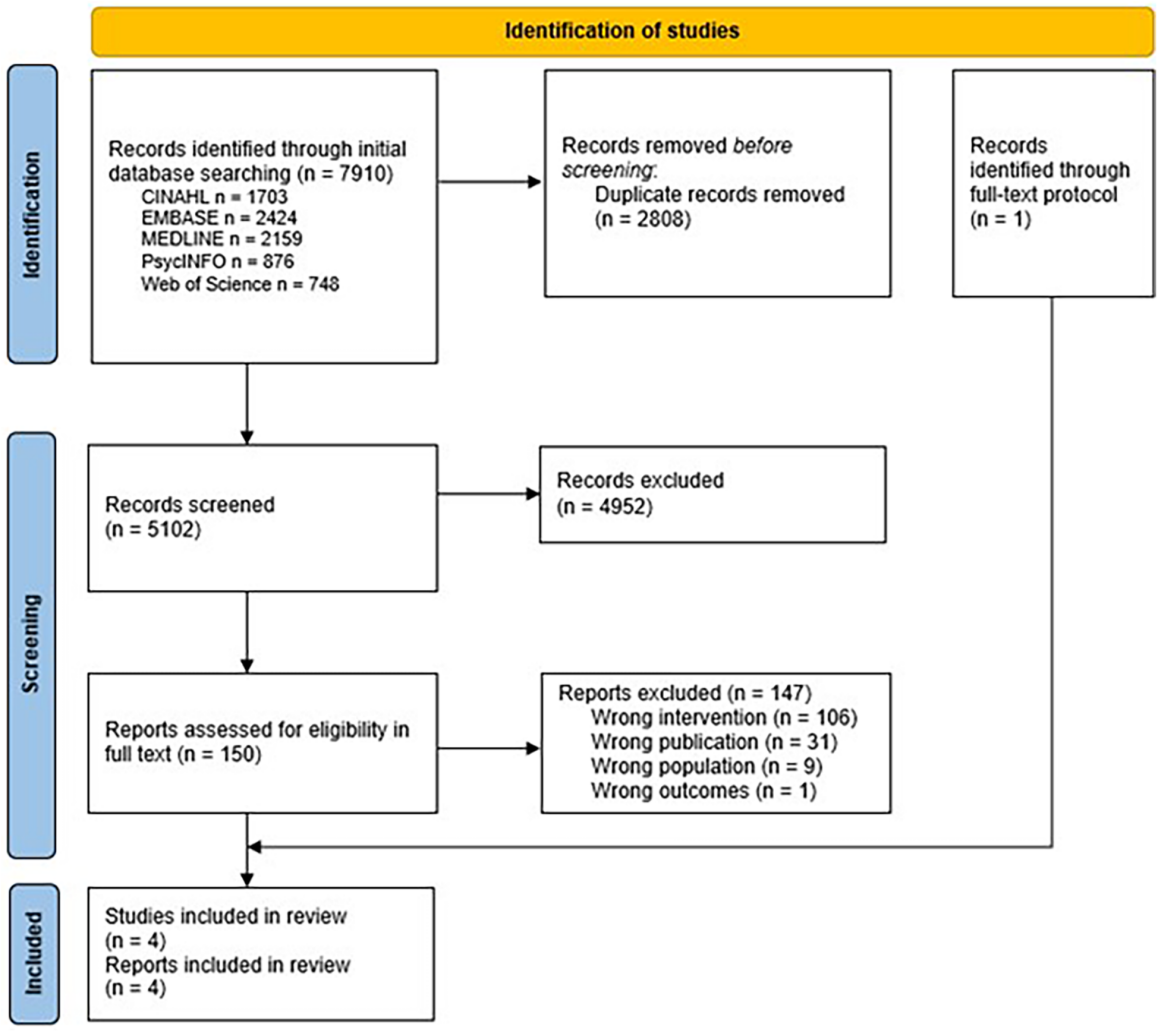
Flowchart of the screening process.

Our searches of the seven databases provided 7,910 records, of which 2,808 were duplicates (Figure 1). We screened the titles and abstracts of 5,102 records of which 4,952 were excluded. Our search identified one research protocol (43) not identified in our search, which we excluded to the benefit of the full report (44). Ultimately, four reports (44–47) from four unique studies were included in the review. A complete list of excluded full-text articles (*n* = 147) and the reason for exclusion are provided in the Supplementary File S3.

### Characteristics of the included studies

The included reports were published between 2017 and 2022 (Table 2). Three reports were conducted in the US (44, 46, 47) and one in Europe (45). In total, 402 children were enrolled across the studies. Zoellner et al. (43) enrolled parent-child dyads (*n* = 66) in one treatment group and parents only in another treatment group (*n* = 62) for comparison. Hoeeg et al. recruited families (*n* = 21) (44), while Yuhas et al. enrolled parents only (*n* = 94) (47). Two studies (44, 46) described the effect of the interventions, while the remaining two used different methods to report experiences from the interventions (45, 47). Three studies employed quantitative designs, i.e., two employed RCT designs, and one employed a non-randomized descriptive design (44, 46, 47), while Hoeeg et al. conducted qualitative interviews (45). We noticed an overlap of authors in the studies by Zoellner and Yuhas (43, 46). Health literacy was measured in three of the studies (44, 46, 47), all using the Newest Vital Sign (NVS) health literacy screening tool. The NVS is based on a nutrition label from an ice cream container. Patients are given the label and asked six questions to be completed in three minutes (48). One study used health literacy as an outcome measure (46).

**Table 2:** Characteristics of the included reports.

First author, year, country	Aim	Design	Study population characteristics and sample size
Hoeeg et al. (45), Denmark	To study whether and how an analytical framework focusing on communicative authenticity can be used to observe and elaborate upon aspects of adherence concerning health behavior change in a concrete family-based intervention.	Qualitative family interviews were analyzed through systematic text condensation.	22 children 11–17 years, BMI above the 99th percentile 39 parents.
Robinson et al. (46), USA	To assess the Stanford GOALS trial—a 3-year, community-based Multi-level, Multi-setting, Multi-component (MMM) systems intervention to reduce weight gain among low socioeconomic status Latinx children with excess weight or obesity.	Two-arm, parallel-group, randomized, open-label, active placebo-controlled trial with blind assessment over three years.	241 children, mean age 9.5 (1.4) years, with BMI ≥ 85th percentile. 241 families, 54% with parents with low health literacy measured with the NVS.
Yuhas et al. (47), USA	To evaluate the Teach-Back/Teach-to-Goal (TB/TTG) strategies integrated within support calls were delivered to parents as part of a 3-month intervention for children with excess weight or obesity.	Secondary analysis based on a pilot feasibility study.	94 parents of children between 8 and 12 years with excess weight or obesity, BMI ≥ 85th percentile. 34% of parents had low health literacy measured with NVS.
Zoellner et al. (44), USA	To compare two 6-month family-based interventions for children with excess weight or obesity in one underserved region of the US.	Randomized controlled trial.	139 children, mean age 10.1 (SD 1.7) years, 30% with excess weight (BMI 85th—<95th percentile), and 70% with obesity (BMI ≥ 95th percentile). 11% of parents had low health literacy measured with NVS.

Two reports examined different aspects of the same interventions:—Zoellner et al. evaluated both “iChoose” and “Family Connections”, while Yuhas et al. solely evaluated the “iChoose” intervention (44, 47) (Table 3). The remaining two presented unique interventions (45, 46). The intervention duration ranged from one single consultation to three years, and all interventions were conducted in collaboration between specialized and community-based care facilities (44–47). Two studies used Teach-Back/Teach-to-Goal (TB/TTG) strategies as part of the intervention, but health literacy was primarily measured at baseline to categorize participants, rather than as an ongoing outcome (44, 47).

**Table 3:** Characteristics of the interventions in the included studies.

Author, year	Theoretical framework	Intervention characteristics	Intervention length	Findings
Hoeeg et al. (45)	Shared care and authenticity approach.	Shared care family-based intervention. Baseline visits to the hospital to obtain the medical/lifestyle history of the child and parents and draw up a collaborative treatment plan to promote necessary lifestyle and behavioral changes and to support the child and family in informing and adjusting the surroundings. Follow-up visits (45 min) every 10th to 12th week by specially trained nurses in the child's local municipality if needed.	If needed, at least one follow-up visit in the municipality every 10–12 weeks. Intervention length was not reported specifically.	The sharing of care adds the potential for several kinds of communicative authenticity because families meet the medical knowledge authority at the hospital and the local nurses in their municipality.
Robinson et al. (46)	Bandura's social cognitive model.	MMM complex systems intervention, with planned interactions, mutual reinforcement, repetition, and positioning complementary elements across the different levels, settings, and components through five modules. Changes in home environment and physical activity after school. Medical information is given based on low health literacy.	Three years.	The MMM intervention did not reduce BMI gain compared to a health education (HE) intervention over 3 years. Effects at 1 and 2 years show the promise of a systems intervention approach. Health literacy improved more in the MMM intervention group than in the HE group.
Yuhas et al. (47)	Health literacy.	*i*Choose family-based intervention. Support calls with TB/TTG health literacy strategies as part of a childhood obesity treatment trial. *i*Choose included (1) bi-weekly family nutrition and exercise sessions; (2) bi-weekly caregiver telephone support calls to set goals, resolve barriers, and reinforce content using TB/TTG strategies between family nutrition and exercise sessions; (3) twice-weekly exercise sessions; (4) workbooks for both parents and children; and (5) children's newsletters to reinforce content.	Three months.	Support calls using TB/TTG strategies were feasible, well received, and should be considered for incorporation into childhood obesity interventions.
Zoellner et al. (44)	Family-based behavior modification.	*i*Choose and Family Connections. *i*Choose is a high-intensity child–parent dyad intervention with 12 family classes, 12 calls using TB/TTG strategies, and 48 exercise sessions. Family Connections is a low-intensity, parent-based intervention with two parent classes and 10 calls.	Six months.	No significant improvement in the child's BMI z-score in either intervention. Relative to *i*Choose, Family Connections had less retention, better management, and lower cost, suggesting low-intensity interventions might be a better fit for the population.

### Methodological appraisal

The methodological quality of the qualitative study (45) and the nonrandomized study was high due to sufficient reporting and adherence to methodological guidelines (47), while the randomized trials (44, 46) had a risk of bias due to a lack of details on blinding, and a lack of complete data and adherence to the intervention (Table 4).

**Table 4:** Methodological quality of the included studies (40).

Qualitative study							
Authors	Are the research questions clear (RQ)?	Does the collected data allow us to address the RQ?	Is the qualitative approach appropriate to answer the RQ?	Is data collection adequate to address the RQ?	Are findings adequately derived from the data?	Is the interpretation of results sufficiently substantiated by the data?	Is there coherence between data, collection, analysis, and interpretation?
Hoeeg et al. (45)	Y	Y	Y	Y	Y	Y	Y
Quantitative randomized controlled trials							
Authors	Clear RQ?	Did the collected data allow us to address the RQ?	Was randomization appropriately performed?	Were the groups comparable at baseline?	Was the outcome data complete?	Were the outcome assessors blinded to the intervention provided?	Did participants adhere to the assigned intervention?
Robinson et al. (46)	Y	Y	Y	Y	N	C	N
Zoellner et al. (44)	Y	Y	C	Y	N	C	N
Quantitative nonrandomized study							
Authors	Was there a clear RQ?	Did the collected data allow us to address the RQ?	Were participants representative of the target population?	Were measurements appropriate regarding the outcome and intervention?	Was the outcome data complete?	Were confounders accounted for in the design and analysis?	During the study, was the intervention administered as intended?
Yuhas et al. (47)	Y	Y	Y	Y	Y	Y	Y

Yes, Y; No, N; Can’t tell, C.

### Synthesis of findings

#### The role of health literacy in the included studies

The four included studies demonstrated varied ways in which health literacy was integrated into childhood obesity interventions, including assessment, tailoring interventions, and evaluation ofeffects. Despite these differences, several common themes emerged about the role and impact of health literacy on intervention effectiveness.

One central theme was the importance of assessing health literacy to understand participant needs. In three studies, health literacy was assessed in parents and measured with the NVS tool, indicating a common recognition of the need to stratify participants by health literacy levels (44, 46, 47). Zoellner et al. (44) and Yuhas et al. (47) only measured parents' health literacy at baseline to categorize parents in low and adequate health literacy. In contrast, Robinson et al. (46) evaluated changes in parental health literacy over time, revealing a favorable increase among participants in the MMM intervention group, suggesting that certain interventions could effectively improve caregiver health literacy (46).

Another recurring theme was the tailoring of communication in intervention strategies based on health literacy. Robinson et al. (46) describe how blood sample results were communicated appropriately for caregiver participants with low health literacy during their intervention. Beyond this, there was no description of whether the intervention was specifically designed to accommodate different health literacy needs among the participants (46).

The effectiveness of TB/TTG strategies featured prominently in two of the studies. In the iChoose intervention investigated by Yuhas et al. (47), healthcare personnel used TB/TTG techniques embedded within support calls about beneficial child health behaviors (nutrition and exercise). The purpose of Yuhas et al.'s study (47) was to evaluate how parents responded to these TB/TTG conversations. The support conversations were well accepted among participants with both low and adequate health literacy, and all appeared to increase their comprehension of key learning outcomes. However, parents with adequate health literacy better understood the content of more support conversations than those with low health literacy (47). In the study by Zoellner et al. (44), the iChoose intervention was compared to the Family Connections intervention. Here, health literacy was measured to describe the intervention groups, but was not included in the analyses.

In the study of Hoeeg et al. (45) the concept of communicative authenticity emerged as imperative in determining how families experienced and applied health information. The authors explored how participants in family-based obesity treatment understood health information, using an analytical framework focused on communicative authenticity or how people can apply health information to their everyday lives. This qualitative study described how families experienced shared care education intervention (45). The authors concluded that the potential of the intervention was unfulfilled. Families who experienced the intervention as authentic found it easier to implement the treatment plan as intended; the authors discussed whether the experience of authenticity was mediated by the family's level of health literacy (45).

In summary, the reviewed studies, particularly the study of Yuhas et al. (47), suggest that adapting treatment plans and interventions to align with the health literacy levels of children and their families may enhance active participation in their care.

## Discussion

There is strikingly little research on health literacy interventions for children with excess weight or obesity, particularly because weight management requires lifelong endeavors. We identified only four studies that met our inclusion criteria. While health literacy is increasingly recognized as a key factor for managing weight in children (20, 21, 23, 26) and is more frequently addressed in prevention studies (49), it has not been fully integrated into the research design of obesity management interventions tailored for children with excess weight and obesity and their parents. The methodological quality of the included studies varied, with a risk of bias in the randomized trials. Only Robinson et al. (46) provided long-term follow-up measures at one and two years; however, their study was limited by the risk of bias. The treatment of obesity requires long-term follow-up; rapid change is unrealistic. Children and parents must be informed about the extended treatment timeline, and measures to increase health literacy should be tailored accordingly, reflecting that health literacy evolves over time with factors such as age, education, and social interactions.

While the overall role of health literacy in interventions for children with excess weight or obesity remains unclear, the included studies revealed some key insights to discuss. Measuring health literacy in parents and children before intervention may serve several important purposes. Three of the included studies assessed parents' level of health literacy at baseline (44, 46, 47). In one study, health literacy was a secondary outcome after the intervention; the intervention was tailored to the participant's health literacy at a functional level when communicating blood test results to the parents (46). The other two studies used only the health literacy scores to categorize the participants (44, 47) but not to inform intervention delivery. Realizing the potential of knowing a family's health literacy needs might offer advantages beyond a more universal approach. Although without effects on BMI reduction, the intervention in the study by Robinson et al. (46) indicated a positive effect on health literacy among those with low health literacy, constituting an example of the potential of tailored interventions. However, research-based knowledge of the interplay between health literacy and other factors known to be associated with different stages of excess weight and obesity is still lacking.

To further expand on the potential of including health literacy in obesity management, our findings indicate a need for more theory-guided interventions tailored to the specific needs of children and their families. There is currently a shortage of theoretical frameworks that link health literacy to health outcomes (50). Studies of adult patient populations have found a relationship between health literacy and health behavior and that the effectiveness of health literacy can be measured by an individual's ability to carry out positive health behaviors (51). However, only a few studies have investigated similar associations in pediatric patient populations, with inconclusive results (51).

None of the included studies assessed children's health literacy directly, likely due to challenges in measuring health literacy in children who have not yet developed strong reading and writing skills. While intellectual development is individual, at this age, children are dependent on their parents and their health literacy. Knowledge on verbal vs. written health literacy among children is scarce. Furthermore, children depend on their parents to make health-related decisions, which is why parents are assessed much more frequently than children. However, studies have demonstrated that children as young as three can actively engage in their healthcare and understand health information when adapted to their developmental level (52, 53). Playful educational interventions supporting health literacy can effectively increase health knowledge and change health behaviors, although it remains a challenge to determine whether effects are retained over time (16).

In one of our reviewed studies, the authors highlighted the challenges of implementing and engaging families in interventions (44). Lower intensity, parent-focused interventions like Family Connections were more practical and effective in a medically underserved region than the more extensive iChoose intervention (44). This finding is consistent with the need to tailor health literacy interventions to the specific needs and resources of the target population, ensuring that they are accessible, engaging, and sustainable (13). Here, using advanced information technology can increase the scalability of health literacy-supportive interventions (54, 55). Still, knowledge about the families' health literacy before an intervention remains important to realize the potential of such solutions (56). While in-person and digital interventions have shown promise for improving health literacy in parents (57), e-health interventions may offer opportunities for treating children and adolescents with excess weight and obesity (58). Regardless of the technology used, Robinson et al. (46) emphasized the importance of culturally tailored communication and education, particularly in low socioeconomic and diverse populations, suggesting that health literacy may mediate the effectiveness of lifestyle interventions, especially in underserved populations (28, 29).

Similarly, Hoegg et al. (45) highlighted that those who did not identify with the intervention or relate to the content were less engaged. Thus, interventions that convey information, enhance the perceived genuineness of health communication, and are well-aligned with the family's values and experiences (44, 45, 47), might improve health outcomes by ensuring that the information provided is understandable, supportive, and actionable (22, 26). Emerging artificial intelligence (AI) technologies create new opportunities for integrating health literacy components in real-time interactive, personalized support for health behavior decisions (55). However, whether AI-based health literacy interventions can offer adaptive, sustainable learning experiences for children with weight or obesity and their families remains to be rigorously tested in well-designed trials.

Previously, health literacy was viewed as a skill or asset that individuals were required to improve. Lately, there has been an increased acknowledgment of the responsibility of healthcare services to meet patients' numerous health literacy needs (59). One commonly used strategy to reassure that the patient has understood and can recall health information is “teach-back”. This technique, recommended as a health literacy communication approach and is often repeated through a “Teach To Goal” (TTG) process to ensure the participants understanding of what is communicated (60), was applied in one intervention subjected to two reports in this review (44, 47). Yuhas et al. (47) demonstrated that using TB/TTG strategies helped bridge the comprehension gap between parents with different health literacy levels, ensuring that all participants, regardless of their initial health literacy, could benefit from the intervention.

This finding supports the use of TB/TTG methods when designing interventions with health literacy elements that could be beneficial in managing childhood obesity (24). However, when Zoellner et al. (44) compared the intervention using TB/TTG with a lower intensity intervention targeting the parents, the latter was more effective. A communicative approach should, therefore, be tailored to the family's level of health literacy as well as their beliefs and values (61). Shared decision making aligns with the broader goals of responsiveness in health literacy interventions by empowering families to make informed, value-based decisions vital for enhancing both health outcomes and patient satisfaction in pediatric obesity management (15, 17).

## Limitations

The major limitation of this review is the low number of included interventions. On the other hand, this limitation reveals a research gap reflected in previous research on health literacy among children (25). Despite a rigorous and exhaustive systematic search across databases, some relevant literature might have been unidentified. Similarly, the small number of studies and the variability in intervention design and implementation limit the generalizability of the findings. Furthermore, during the review process, we identified reports on the same study population and intervention. In such cases, we chose to include the report that provided the most comprehensive and detailed information relevant to our research question.

## Conclusion

Despite the increasing prevalence of childhood obesity and its associated negative health outcomes, surprisingly little attention has been paid to health literacy as potentially important for successful obesity interventions for children. It is crucial to improve families' access to understandable and trustworthy health information and their ability to use it effectively. Health literacy plays a key role in empowering families to make informed health decisions and engage in health-related behaviors, thereby enhancing the likelihood of achieving and maintaining weight reduction. Future research should focus on integrating health literacy into weight management interventions in a systematic and theory-driven manner, ensuring that these interventions are tailored to the specific needs of children and their families.

## Data Availability

The raw data supporting the conclusions of this article will be made available by the authors, without undue reservation.

## Supplementary material

Supplementary File S1 Reporting checklist PRISMA.

Supplementary File S2 Complete overview of search strategies.

Supplementary File S3 Complete list of all excluded full-text studies.

## References

[1] World Health Organization (WHO). Obesity and Overweight. Fact Sheet. (2024). Available online at: https://www.who.int/news-room/fact-sheets/detail/obesity-and-overweight (Accessed October 06, 2024)

[2] World Health Organization (WHO). European Regional Obesity Report 2022. (2022). Available online at: https://www.who.int/europe/publications/i/item/9789289057738 (Accessed October 06, 2023).

[3] Balthasar MR Roelants M Brannsether-Ellingsen B Bjarnason R Bergh IH Kvalvik LG Trends in overweight and obesity in Bergen, Norway, using data from routine child healthcare 2010–2022. Acta Paediatr. (2024). 38895765 10.1111/apa.17323

[4] Mekonnen T Papadopoulou E Arah OA Brantsæter AL Lien N Gebremariam MK. Socioeconomic inequalities in Children’s weight, height and BMI trajectories in Norway. Sci Rep. (2021) 11(1):4979. 10.1038/s41598-021-84615-w 33654136 PMC7925535

[5] Jebeile H Kelly AS O'Malley G Baur LA. Obesity in children and adolescents: epidemiology, causes, assessment, and management. Lancet Diabetes Endocrinol. (2022) 10(5):351–65. 10.1016/S2213-8587(22)00047-X 35248172 PMC9831747

[6] Ng M Fleming T Robinson M Thomson B Graetz N Margono C Global, regional, and national prevalence of overweight and obesity in children and adults during 1980–2013: a systematic analysis for the global burden of disease study 2013. Lancet. (2014) 384(9945):766–81. 10.1016/S0140-6736(14)60460-8 24880830 PMC4624264

[7] Griffiths LJ Parsons TJ Hill AJ. Self-esteem and quality of life in obese children and adolescents: a systematic review. Int J Pediatr Obes. (2010) 5(4):282–304. 10.3109/17477160903473697 20210677

[8] Pulgarón ER. Childhood obesity: a review of increased risk for physical and psychological comorbidities. Clin Ther. (2013) 35(1):A18–32. 10.1016/j.clinthera.2012.12.014 23328273 PMC3645868

[9] Simmonds M Burch J Llewellyn A Griffiths C Yang H Owen C The use of measures of obesity in childhood for predicting obesity and the development of obesity-related diseases in adulthood: a systematic review and meta-analysis. Health Technol Assess. (2015) 19(43):1–336. 10.3310/hta19430 26108433 PMC4781104

[10] Nordic Council of Ministers (2023) Nordic Nutrition Recommendations 2023. Integrating Environmental Aspects. (2024) 113:2098–106. 10.1111/apa.17323

[11] Gray LA Hernandez Alava M Kelly MP Campbell MJ. Family lifestyle dynamics and childhood obesity: evidence from the millennium cohort study. BMC Public Health. (2018) 18(1):500. 10.1186/s12889-018-5398-5 29807535 PMC5971431

[12] Ho M Garnett SP Baur L Burrows T Stewart L Neve M Effectiveness of lifestyle interventions in child obesity: systematic review with meta-analysis. Pediatrics. (2012) 130(6):e1647–71. 10.1542/peds.2012-1176 23166346

[13] Kirk S Ogata B Wichert E Handu D Rozga M. Treatment of pediatric overweight and obesity: position of the academy of nutrition and dietetics based on an Umbrella review of systematic reviews. J Acad Nutr Diet. (2022) 122(4):848–61. 10.1016/j.jand.2022.01.008 35063666

[14] Hampl SE Hassink SG Skinner AC Armstrong SC Barlow SE Bolling CF Clinical practice guideline for the evaluation and treatment of children and adolescents with obesity. Pediatrics. (2023) 151(2):e2022060640. 36622115 10.1542/peds.2022-060640

[15] Cardel MI Atkinson MA Taveras EM Holm J-C Kelly AS. Obesity treatment among adolescents: a review of current evidence and future directions. JAMA Pediatr. (2020) 174(6):609–17. 10.1001/jamapediatrics.2020.0085 32202626 PMC7483247

[16] Ribeiro SM Basso MB Massignan C Leal SC. Playful educational interventions in children and Adolescents’ health literacy: a systematic review. Health Promot Int. (2023) 38(4):daad089. 10.1093/heapro/daad089 37647524

[17] Steinbeck KS Lister NB Gow ML Baur LA. Treatment of adolescent obesity. Nat Rev Endocrinol. (2018) 14(6):331–44. 10.1038/s41574-018-0002-8 29654249

[18] Wahl AK Andersen MH Ødemark J Reisaether A Urstad KH Engebretsen E. The importance of shared meaning-making for sustainable knowledge translation and health literacy. J Eval Clin Pract. (2022) 28(5):828–34. 10.1111/jep.13690 35466469 PMC9790374

[19] World Health Organization (WHO). Regional Office for South-East Asia. Health Literacy Toolkit for Low- and Middle-Income Countries: A Series of Information Sheets to Empower Communities and Strengthen Health Systems. Melbourne, Australia: WHO Regional Office for South-East Asia (2015). Available online at: https://iris.who.int/handle/10665/205244 (Accessed October 06, 2024).

[20] Sharif I Blank AE. Relationship between child health literacy and body mass Index in overweight children. Patient Educ Couns. (2010) 79(1):43–8. 10.1016/j.pec.2009.07.035 19716255 PMC2839034

[21] Chari R Warsh J Ketterer T Hossain J Sharif I. Association between health literacy and child and adolescent obesity. Patient Educ Couns. (2014) 94(1):61–6. 10.1016/j.pec.2013.09.006 24120396

[22] Morrison AK Glick A Yin HS. Health literacy: implications for child health. Pediatr Rev. (2019) 40(6):263–77. 10.1542/pir.2018-0027 31152099

[23] Chrissini MK Panagiotakos DB. Health literacy as a determinant of childhood and adult obesity: a systematic review. Int J Adolesc Med Health. (2021) 33(3):9–39. 10.1515/ijamh-2020-0275 33592684

[24] White RO Thompson JR Rothman RL McDougald Scott AM Heerman WJ Sommer EC A health literate approach to the prevention of childhood overweight and obesity. Patient Educ Couns. (2013) 93(3):612–8. 10.1016/j.pec.2013.08.010 24001660 PMC3904952

[25] Bröder J Okan O Bauer U Bruland D Schlupp S Bollweg TM Health literacy in childhood and youth: a systematic review of definitions and models. BMC Public Health. (2017) 17(1):361. 10.1186/s12889-017-4267-y 28441934 PMC5405535

[26] Fleary SA Joseph P Pappagianopoulos JE. Adolescent health literacy and health behaviors: a systematic review. J Adolesc. (2018) 62:116–27. 10.1016/j.adolescence.2017.11.010 29179126

[27] Nutbeam D Kickbusch I. Health promotion glossary. Health Promot Int. (1998) 13(4):349–64. 10.1093/heapro/13.4.349

[28] Delbosq S Velasco V Vercesi C Lombardia GRH Vecchio LP. Adolescents’ nutrition: the role of health literacy, family and socio-demographic variables. Int J Environ Res Public Health. (2022) 19(23):15719. 10.3390/ijerph192315719 36497794 PMC9736019

[29] Bann D Johnson W Li L Kuh D Hardy R. Socioeconomic inequalities in childhood and adolescent body-mass index, weight, and height from 1953 to 2015: an analysis of four longitudinal, observational, British birth cohort studies. Lancet Public Health. (2018) 3(4):e194–203. 10.1016/S2468-2667(18)30045-8 29571937 PMC5887082

[30] Libuy N Bann D Fitzsimons E. Inequalities in body mass index, diet and physical activity in the UK: longitudinal evidence across childhood and adolescence. SSM Popul Health. (2021) 16:100978. 10.1016/j.ssmph.2021.100978 34950761 PMC8671115

[31] Kebbe M Perez A Buchholz A McHugh T-LF Scott SD Richard C Conversation cards for adolescents©: a patient-centered communication and behavior change tool for adolescents with obesity and health care providers. J Commun Healthc. (2020) 13(2):79–88. 10.1080/17538068.2020.1765126

[32] Zoellner J. The influence of parental health literacy status on reach, attendance, retention, and outcomes in a family-based childhood obesity treatment program, Virginia, 2013–2015. Prev Chronic Dis. (2017) 14:E87. 10.5888/pcd14.160421 28957032 PMC5621521

[33] Aromataris E Lockwood C Porritt K Pilla B Jordan Z, editors. JBI Manual for Evidence Synthesis. Adelaide: JBI (2024). 10.46658/JBIMES-24-01

[34] Pluye P Hong QN. Combining the power of stories and the power of numbers: mixed methods research and mixed studies reviews. Annu Rev Public Health. (2014) 35:29–45. 10.1146/annurev-publhealth-032013-182440 24188053

[35] Moher D Liberati A Tetzlaff J Altman DG Group P. Preferred reporting items for systematic reviews and meta-analyses: the prisma statement. PLoS Med. (2009) 6(7):e1000097. 10.1371/journal.pmed.1000097 19621072 PMC2707599

[36] Peters MDJ Godfrey C McInerney P Munn Z Tricco AC Khalil H. Scoping reviews. In: Aromataris E Lockwood C Porritt K Pilla B Jordan Z, editors. JBI Manual for Evidence Synthesis. JBI (2024). 10.46658/JBIMES-24-09

[37] Cole TJ Bellizzi MC Flegal KM Dietz WH. Establishing a standard definition for child overweight and obesity worldwide: international survey. Br Med J. (2000) 320(7244):1240–3. 10.1136/bmj.320.7244.1240 10797032 PMC27365

[38] McGowan J Sampson M Salzwedel DM Cogo E Foerster V Lefebvre C. Press peer review of electronic search strategies: 2015 guideline statement. J Clin Epidemiol. (2016) 75:40–6. 10.1016/j.jclinepi.2016.01.021 27005575

[39] Veritas-Health-Innovation. Covidence Systematic Review Software. Melbourne, Australia (2020). Available online at: www.covidence.org (Accessed October 06, 2024).

[40] Hong QN Fàbregues S Bartlett G Boardman F Cargo M Dagenais P The mixed methods appraisal tool (mmat) version 2018 for information professionals and researchers. Educ Inf. (2018) 34(4):285–91. 10.3233/EFI-180221

[41] Guyatt GH Oxman AD Vist GE Kunz R Falck-Ytter Y Alonso-Coello P Grade: an emerging consensus on rating quality of evidence and strength of recommendations. Br Med J. (2008) 336(7650):924. 10.1136/bmj.39489.470347.AD 18436948 PMC2335261

[42] Thomas J Harden A. Methods for the thematic synthesis of qualitative research in systematic reviews. BMC Med Res Methodol. (2008) 8(1):1–10. 10.1186/1471-2288-8-45 18616818 PMC2478656

[43] Brito FA Zoellner JM Hill J You W Alexander R Hou X From bright bodies to ichoose: using a Cbpr approach to develop childhood obesity intervention materials for rural Virginia. Sage Open. (2019) 9(1):1–14. 10.1177/2158244019837313 34290901 PMC8291387

[44] Zoellner JY W Hill JL Brock D-JP Yuhas M Price B Wilson J Comparing two different family-based childhood obesity treatment programmes in a medically underserved region: effectiveness, engagement and implementation outcomes from a randomized controlled trial. Pediatr Obes. (2022) 17(1):e12840. 10.1111/ijpo.12840 34396714

[45] Hoeeg D Mortil AMA Hansen ML Teilmann GK Grabowski D. Families’ adherence to a family-based childhood obesity intervention: a qualitative study on perceptions of communicative authenticity. Health Commun. (2020) 35(1):110–8. 10.1080/10410236.2018.1545335 30444139

[46] Robinson TN Matheson D Wilson DM Weintraub DL Banda JA McClain A A community-based, multi-level, multi-setting, multi-component intervention to reduce weight gain among low socioeconomic Status latinx children with overweight or obesity: the stanford goals randomised controlled trial. Lancet Diabetes Endocrinol. (2021) 9(6):336–49. 10.1016/S2213-8587(21)00084-X 33933181 PMC8241238

[47] Yuhas M Zoellner J Hou X Alexander R Hill J You W Understanding teach-back and teach-to-goal strategies embedded in support calls for a health literacy-sensitive childhood obesity treatment trial. Health Lit Res Pract. (2021) 5(3):e208–e17. 34379548 10.3928/24748307-20210713-01 PMC8356482

[48] Weiss BD Mays MZ Martz W Castro KM DeWalt DA Pignone MP Quick assessment of literacy in primary care: the newest vital sign. Ann Fam Med. (2005) 3(6):514–22. 10.1370/afm.405 16338915 PMC1466931

[49] Sanders LM Perrin EM Yin HS Delamater AM Flower KB Bian A A health-literacy intervention for early childhood obesity prevention: a cluster-randomized controlled trial. Pediatrics. (2021) 147(5):e2020049866. 10.1542/peds.2020-049866 33911032 PMC8086006

[50] Walters R Leslie SJ Polson R Cusack T Gorely T. Establishing the efficacy of interventions to improve health literacy and health behaviours: a systematic review. BMC Public Health. (2020) 20(1):1040. 10.1186/s12889-020-08991-0 32605608 PMC7329558

[51] Riemann L Lubasch JS Heep A Ansmann L. The role of health literacy in health behavior, health service use, health outcomes, and empowerment in pediatric patients with chronic disease: a systematic review. Int J Environ Res Public Health. (2021) 18(23):12464. 34886185 10.3390/ijerph182312464 PMC8656602

[52] Stålberg A Sandberg A Söderbäck M. Younger Children’s (three to five years) perceptions of being in a health-care situation. Early Child Dev Care. (2016) 186(5):832–44. 10.1080/03004430.2015.1064405

[53] Derwig M Tiberg I Hallström I. Elucidating the Child’s perspective in health promotion: children’s experiences of child-centred health dialogue in Sweden. Health Promot Int. (2021) 36(2):363–73. 10.1093/heapro/daaa060 32620968 PMC8049549

[54] Holmberg C Berg C Dahlgren J Lissner L Chaplin JE. Health literacy in a complex digital media landscape: pediatric obesity Patients’ experiences with online weight, food, and health information. Health Informatics J. (2019) 25(4):1343–57. 10.1177/1460458218759699 29499615

[55] Fitzpatrick PJ. Improving health literacy using the power of digital communications to achieve better health outcomes for patients and practitioners. Front Digit Health. (2023) 5:1264780. 10.3389/fdgth.2023.1264780 38046643 PMC10693297

[56] Schapira MM Swartz S Ganschow PS Jacobs EA Neuner JM Walker CM Tailoring educational and behavioral interventions to level of health literacy: a systematic review. MDM Policy Pract. (2017) 2(1):2381468317714474. 10.1177/2381468317714474 30288424 PMC6124923

[57] Belfrage SL Husted M Fraser SDS Patel S Faulkner JA. A systematic review of the effectiveness of community-based interventions aimed at improving health literacy of parents/carers of children. Perspect Public Health. (2023):17579139231180746. 10.1177/17579139231180746 37381897 PMC11800687

[58] Azevedo LB Stephenson J Ells L Adu-Ntiamoah S DeSmet A Giles EL The effectiveness of E-health interventions for the treatment of overweight or obesity in children and adolescents: a systematic review and meta-analysis. Obes Rev. (2022) 23(2):e13373. 10.1111/obr.13373 34747118

[59] Trezona A Dodson S Osborne RH. Development of the organisational health literacy responsiveness (org-HLR) self-assessment tool and process. BMC Health Serv Res. (2018) 18(1):694. 10.1186/s12913-018-3499-6 30189874 PMC6128002

[60] Talevski J Wong Shee A Rasmussen B Kemp G Beauchamp A. Teach-back: a systematic review of implementation and impacts. PLoS One. (2020) 15(4):e0231350. 10.1371/journal.pone.0231350 32287296 PMC7156054

[61] Moore J Haemer M Mirza N Z Weatherall Y Han J Mangarelli C Pilot testing of a patient decision aid for adolescents with severe obesity in us pediatric weight management programs within the compass network. Int J Environ Res Public Health. (2019) 16(10):20. 10.3390/ijerph16101776 PMC6572315 31137491

