# Supplementary material for: The role of health literacy in intervention studies targeting children living with overweight or obesity and their parents—a systematic mixed methods review
Source: Front Pediatr. 2025 Jan 22;12:1507379. doi: 10.3389/fped.2024.1507379 (PMC11794496; doi:10.3389/fped.2024.1507379)
Supplement: Supplementary File S2 [file Supplementaryfile2.docx]

**Database**: Ovid MEDLINE(R) and Epub Ahead of Print, In-Process, In-Data-Review & Other Non-Indexed Citations and Daily <1946 to October 25, 2023>Ovid

| **#** | **Searches October 23^rd^, 2023** |
| --- | --- |
| 1 | ((Child* or infant* or toddler* or kid* or boy* or girl* or schoolchild* or preschooler* or juvenile* or preteen* or teen* or adolescen* or preadolescen* or youth or young people or young person* or prepuberty or puberty or minor or minors or early age or paediatric* or pediatric*) adj4 (Obese or obesit* or adipose or adiposit* or overweight* or over weight* or body weight* or bodyweight* or BMI or body mass or body size* or waist circumference* or body fat* or fatness or (weight adj1 (change* or gain* or los* or reduc* or manag* or control* or maint* or variation* or disorder*)))).tw,kw,kf. |
| 2 | child/ or infant/ |
| 3 | adolescent/ or puberty/ or Minors/ |
| 4 | Pediatrics/ |
| 5 | child, preschool/ |
| 6 | 1 or 2 or 3 or 4 or 5 |
| 7 | overweight/ or body weight/ |
| 8 | obesity/ or obesity hypoventilation syndrome/ or obesity, abdominal/ or obesity, metabolically benign/ or obesity, morbid/ or pediatric obesity/ |
| 9 | "body weights and measures"/ |
| 10 | Body Mass Index/ or body fat distribution/ or body size/ or waist-height ratio/ |
| 11 | adiposity/ |
| 12 | body weight changes/ or weight gain/ or weight loss/ or Weight Reduction Programs/ |
| 13 | ((Child* or infant* or toddler* or kid* or boy* or girl* or schoolchild* or preschooler* or juvenile* or preteen* or teen* or adolescen* or preadolescen* or youth or young people or young person* or prepuberty or puberty or minor or minors or early age or paediatric* or pediatric*) adj4 (Obese or obesit* or adipose or adiposit* or overweight* or over weight* or body weight* or bodyweight* or BMI or body mass or body size* or waist circumference* or body fat* or fatness or (weight adj1 (change* or gain* or los* or reduc* or manag* or control* or maint* or variation* or disorder*)))).tw,kw,kf. |
| 14 | 7 or 8 or 9 or 10 or 11 or 12 or 13 |
| 15 | (health adj2 (literac* or belief* or attitude* or competenc* or knowledge or understand* or comprehen* or communicat* or teaching or coaching or counseling or decision* or skill* or proficien* or adept* or mastery or education*)).tw,kw,kf. |
| 16 | ((physical* or nutrit* or food or diet*) adj2 literac*).tw,kw,kf. |
| 17 | Health Literacy/ |
| 18 | health knowledge, attitudes, practice/ or attitude to health/ or health communication/ |
| 19 | Health Education/ |
| 20 | 15 or 16 or 17 or 18 or 19 |
| 21 | 6 and 14 |
| 22 | 20 and 21 |
| 23 | (intervention* or program* or activit* or training* or course* or approach or approaches).tw,kw,kf. |
| 24 | 22 and 23 |
| 25 | limit 24 to yr="2013 -Current" |

**Database**: Embase <1974 to 2023 Week 42> via Ovid

| **#** | **Searches October 26^th^, 2023** |
| --- | --- |
| 1 | child/ or juvenile/ or boy/ or girl/ or infant/ or preschool child/ or school child/ or toddler/ |
| 2 | puberty/ or adolescence/ or prepuberty/ or "minor (person)"/ |
| 3 | pediatrics/ |
| 4 | ((Child* or infant* or toddler* or kid* or boy* or girl* or schoolchild* or preschooler* or juvenile* or preteen* or teen* or adolescen* or preadolescen* or youth or young people or young person* or prepuberty or puberty or minor or minors or early age or paediatric* or pediatric*) adj4 (Obese or obesit* or adipose or adiposit* or overweight* or over weight* or body weight* or bodyweight* or BMI or body mass or body size* or waist circumference* or body fat* or fatness or (weight adj1 (change* or gain* or los* or reduc* or manag* or control* or maint* or variation* or disorder*)))).tw,kw,kf. |
| 5 | 1 or 2 or 3 or 4 |
| 6 | body weight/ or body weight change/ or body weight control/ or ideal body weight/ |
| 7 | body weight gain/ or body weight variation/ or body weight management/ or body weight maintenance/ |
| 8 | weight loss program/ or obesity management/ or obesity/ or abdominal obesity/ or adolescent obesity/ or childhood obesity/ or morbid obesity/ or normal weight obesity/ |
| 9 | waist hip ratio/ or obesity hypoventilation syndrome/ or childhood obesity intervention/ |
| 10 | body mass/ |
| 11 | body fat/ or body fat distribution/ |
| 12 | waist to height ratio/ or waist circumference/ |
| 13 | ((Child* or infant* or toddler* or kid* or boy* or girl* or schoolchild* or preschooler* or juvenile* or preteen* or teen* or adolescen* or preadolescen* or youth or young people or young person* or prepuberty or puberty or minor or minors or early age or paediatric* or pediatric*) adj4 (Obese or obesit* or adipose or adiposit* or overweight* or over weight* or body weight* or bodyweight* or BMI or body mass or body size* or waist circumference* or body fat* or fatness or (weight adj1 (change* or gain* or los* or reduc* or manag* or control* or maint* or variation* or disorder*)))).tw,kw,kf. |
| 14 | 6 or 7 or 8 or 9 or 10 or 11 or 12 or 13 |
| 15 | health literacy/ or health education/ |
| 16 | attitude to health/ |
| 17 | (health adj2 (literac* or belief* or attitude* or competenc* or knowledge or understand* or comprehen* or communicat* or teaching or coaching or counseling or decision* or skill* or proficien* or adept* or mastery or education*)).tw,kw,kf. |
| 18 | ((physical* or nutrit* or food or diet*) adj2 literac*).tw,kw,kf. |
| 19 | or/15-18 |
| 20 | (intervention* or program* or activit* or training* or course* or approach or approaches).tw,kw,kf. |
| 21 | 5 and 14 and 19 and 20 |
| 22 | limit 21 to yr="2013 -Current" |

**Database**: APA PsycInfo <1806 to October Week 3 2023> via Ovid

| **#** | **Searches October 26^th^, 2023** |
| --- | --- |
| 1 | ("100" or "140" or "160" or "180" or "200").ag. |
| 2 | puberty/ or early adolescence/ or pediatrics/ |
| 3 | ((Child* or infant* or toddler* or kid* or boy* or girl* or schoolchild* or preschooler* or juvenile* or preteen* or teen* or adolescen* or preadolescen* or youth or young people or young person* or prepuberty or puberty or minor or minors or early age or paediatric* or pediatric*) adj4 (Obese or obesit* or adipose or adiposit* or overweight* or over weight* or body weight* or bodyweight* or BMI or body mass or body size* or waist circumference* or body fat* or fatness or (weight adj1 (change* or gain* or los* or reduc* or manag* or control* or maint* or variation* or disorder*)))).tw. |
| 4 | 1 or 2 or 3 |
| 5 | Body Weight/ or overweight/ or obesity/ |
| 6 | body mass index/ or body fat/ or body size/ or weight control/ or weight gain/ or weight loss/ |
| 7 | ((Child* or infant* or toddler* or kid* or boy* or girl* or schoolchild* or preschooler* or juvenile* or preteen* or teen* or adolescen* or preadolescen* or youth or young people or young person* or prepuberty or puberty or minor or minors or early age or paediatric* or pediatric*) adj4 (Obese or obesit* or adipose or adiposit* or overweight* or over weight* or body weight* or bodyweight* or BMI or body mass or body size* or waist circumference* or body fat* or fatness or (weight adj1 (change* or gain* or los* or reduc* or manag* or control* or maint* or variation* or disorder*)))).tw. |
| 8 | 5 or 6 or 7 |
| 9 | health literacy/ or health education/ or health knowledge/ |
| 10 | Health Attitudes/ or Health Behavior/ |
| 11 | (health adj2 (literac* or belief* or attitude* or competenc* or knowledge or understand* or comprehen* or communicat* or teaching or coaching or counseling or decision* or skill* or proficien* or adept* or mastery or education*)).tw. |
| 12 | ((physical* or nutrit* or food or diet*) adj2 literac*).tw. |
| 13 | 9 or 10 or 11 or 12 |
| 14 | (intervention* or program* or activit* or training* or course* or approach or approaches).tw. |
| 15 | 4 and 8 and 13 and 14 |
| 16 | limit 15 to yr="2013 -Current" |

**Database**: Cinahl via EbscoHost

| **#** | **Query October 26^th^, 2023** |
| --- | --- |
| S1 | (MH "Child") OR (MH "Child, Preschool") |
| S2 | (MH "Infant") |
| S3 | (MH "Adolescence") |
| S4 | (MH "Puberty") |
| S5 | (MH "Pediatrics") |
| S6 | TI ( (( Child* or infant* or toddler* or kid* or boy* or girl* or schoolchild* preschooler* or "pre schooler*" or juvenile* or preteen* or “pre teen*” or teen* or adolescen* or preadolescen* or “pre adolescen*” or youth or "young people” or “young person*” or prepuberty or “pre puberty*” or puberty or minor or minors or "early age" or paediatric* or pediatric*) N3 ((Obese or obesit* or adipose or adiposit* or overweight* or “over weight*” or “body weight*” or bodyweight* or BMI or “body mass” or “body size*” or “waist circumference*” or “body fat*” or fatness or ((weight N0 (change* or gain* or los* or reduc* or manag* or control* or maint* or variation* or disorder*)))) ) OR AB ( (( Child* or infant* or toddler* or kid* or boy* or girl* or schoolchild* or juvenile* or preteen* or “pre teen*” or teen* or adolescen* or preadolescen* or “pre adolescen*” or youth or "young people” or “young person*” or prepuberty or “pre puberty*” or puberty or minor or minors or "early age" or paediatric* or pediatric*) N3 ((Obese or obesit* or adipose or adiposit* or overweight* or “over weight*” or “body weight*” or bodyweight* or BMI or “body mass” or “body size*” or “waist circumference*” or “body fat*” or fatness or ((weight N0 (change* or gain* or los* or reduc* or manag* or control* or maint* or variation* or disorder*)))) ) |
| S7 | S1 OR S2 OR S3 OR S4 OR S5 OR S6 |
| S8 | (MH "Obesity") OR (MH "Pediatric Obesity") OR (MH "Obesity, Morbid") OR (MH "Obesity Paradox") |
| S9 | (MH "Body Weight") OR (MH "Body Weight Changes") OR (MH "Weight Loss") OR (MH "Weight Gain") OR (MH "Weight Reduction Programs") |
| S10 | (MH "Body Mass Index") OR (MH "Waist-Hip Ratio") OR (MH "Waist Circumference") OR (MH "Adipose Tissue Distribution") |
| S11 | TI ( (( Child* or infant* or toddler* or kid* or boy* or girl* or schoolchild* preschooler* or "pre schooler*" or juvenile* or preteen* or “pre teen*” or teen* or adolescen* or preadolescen* or “pre adolescen*” or youth or "young people” or “young person*” or prepuberty or “pre puberty*” or puberty or minor or minors or "early age" or paediatric* or pediatric*) N3 ((Obese or obesit* or adipose or adiposit* or overweight* or “over weight*” or “body weight*” or bodyweight* or BMI or “body mass” or “body size*” or “waist circumference*” or “body fat*” or fatness or ((weight N0 (change* or gain* or los* or reduc* or manag* or control* or maint* or variation* or disorder*)))) ) OR AB ( (( Child* or infant* or toddler* or kid* or boy* or girl* or schoolchild* or juvenile* or preteen* or “pre teen*” or teen* or adolescen* or preadolescen* or “pre adolescen*” or youth or "young people” or “young person*” or prepuberty or “pre puberty*” or puberty or minor or minors or "early age" or paediatric* or pediatric*) N3 ((Obese or obesit* or adipose or adiposit* or overweight* or “over weight*” or “body weight*” or bodyweight* or BMI or “body mass” or “body size*” or “waist circumference*” or “body fat*” or fatness or ((weight N0 (change* or gain* or los* or reduc* or manag* or control* or maint* or variation* or disorder*)))) ) |
| S12 | S8 OR S9 OR S10 OR S11 |
| S13 | (MH "Health Literacy") |
| S14 | (MH "Health Education") |
| S15 | (MH "Health Knowledge") |
| S16 | (MH "Attitude to Health") |
| S17 | (MH "Health Beliefs") |
| S18 | TI ( (health N1 (literac* or belief* or attitude* or competenc* or knowledge or understand* or comprehen* or communicat* or teaching or coaching or counseling or decision* or skill* or proficien* or adept* or mastery)) ) OR AB ( (health N1 (literac* or belief* or attitude* or competenc* or knowledge or understand* or comprehen* or communicat* or teaching or coaching or counseling or decision* or skill* or proficien* or adept* or mastery)) ) |
| S19 | TI ( ((physical* or nutrit* or food or diet*) N1 literac*) ) OR AB ( ((physical* or nutrit* or food or diet*) N1 literac*) ) |
| S20 | S13 OR S14 OR S15 OR S16 OR S17 OR S18 OR S19 |
| S21 | S7 AND S12 AND S20 |
| S22 | S7 AND S12 AND S20 Limiters - Published Date: 20130101-20231231  Search modes - Boolean/Phrase |

Database: Web of Science Core collection

|  | **October 26^th^, 2023** |
| --- | --- |
| #1 | **TS=((((( Child* or infant* or toddler* or kid* or boy* or girl* or schoolchild* or juvenile* or preteen* or teen* or adolescen* or preadolescen* or youth or "young people" or "young person*" or prepuberty or puberty or minor or minors or "early age" or paediatric* or pediatric*)) NEAR/3 (Obese or obesit* or adipose or adiposit* or overweight* or "over weight*" or "body weight*" or bodyweight* or BMI or "body mass" or "body size*" or "waist circumference*" or "body fat*" or fatness or (weight NEAR/0 (change* or gain* or loss or reduction* or manag* or control* or maint* or variation* or disorder*))))))** |
| #2 | TS=( (((health NEAR/1 (literac* or belief* or attitude* or competenc* or knowledge or understand* or comprehen* or communicat* or teaching or coaching or counseling or decision* or skill* or proficien* or adept* or mastery or education*)) OR ((physical* or nutrit* or food or diet*) NEAR/1 literac*)))) |
| #3 | **TS=(intervention* or program* or activit* or training* or course* or approach or approaches)** |
| #4 | **#1 AND #2 AND #3 (avgrenset 2013-2023)** |
