# Supplementary material for: The role of health literacy in intervention studies targeting children living with overweight or obesity and their parents—a systematic mixed methods review
Source: Front Pediatr. 2025 Jan 22;12:1507379. doi: 10.3389/fped.2024.1507379 (PMC11794496; doi:10.3389/fped.2024.1507379)
Supplement: Supplementary File S3 [file Supplementaryfile3.docx]

**Excluded studies**

**Wrong intervention N=106**

| **Authors; DOI** |
| --- |
| **Abdeyazdan, Z.; Moshgdar, H.; Golshiri, P.** [**https://dx.doi.org/10.4103/1735-9066.208163**](https://dx.doi.org/10.4103/1735-9066.208163) |
| **Akgul Gundogdu, N.; Sevig, E. U.; Guler, N.** [**https://dx.doi.org/10.1111/jocn.14246**](https://dx.doi.org/10.1111/jocn.14246) |
| Amini, M.; Djazayery, A.; Majdzadeh, R.; Taghdisi, M. H.; Sadrzadeh-Yeganeh, H.; Abdollahi, Z.; Hosseinpour-Niazi, N.; Chamari, M.; Nourmohammadi, M. <https://dx.doi.org/10.1177/1099800416654261> |
| Arauz Boudreau, A. D.; Kurowski, D. S.; Gonzalez, W. I.; Dimond, M. A.; Oreskovic, N. M. <https://dx.doi.org/10.1016/j.amepre.2012.11.026> |
| Archuleta, Martha; VanLeeuwen, Dawn; Turner, Carol <https://dx.doi.org/10.1016/j.jneb.2016.03.012> |
| Azrin Shah, A. B.; Aishath, N.; Al Oran, H. M.; Hani Farhana, N.; Azreena, M. B.; Fatima Dahiru, M.; Saba Babeli, Y.; Suwanmanee, S.; Hassan, I.; Alsharif Mohammed, K.; Sahar Saeed, B.; Mohamed Osman, A.; Suriani, I.; Ahmad Iqmer Nashriq, M. N.; NorAfiah, M. Z.; Rosliza, A. M. |
| Bagherniya, M.; Sharma, M.; Mostafavi Darani, F.; Maracy, M. R.; Safarian, M.; Allipour Birgani, R.; Bitarafan, V.; Keshavarz, S. A. <https://dx.doi.org/10.1177/0272684X17749566> |
| Bala, N.; Price, S. N.; Horan, C. M.; Gerber, M. W.; Taveras, E. M. <https://dx.doi.org/10.1177/0009922819837371> |
| Bani Salameh, A.; Al-Sheyab, N.; El-Hneiti, M.; Shaheen, A.; Williams, L. M.; Gallagher, R. <https://dx.doi.org/10.1111/ijn.12528> |
| Berkowitz, R. I.; Rukstalis, M. R.; Bishop-Gilyard, C. T.; Moore, R. H.; Gehrman, C. A.; Xanthopoulos, M. S.; Cochran, W. J.; Louden, D.; Wadden, T. A. 10.1093/jpepsy/jst035 |
| Boles, R. E.; Yun, L.; Hambidge, S. J.; Davidson, A. <https://dx.doi.org/10.1177/0009922815570614> |
| Boutelle, K. N.; Braden, A.; Douglas, J. M.; Rhee, K. E.; Strong, D.; Rock, C. L.; Wilfley, D. E.; Epstein, L.; Crow, S. <https://dx.doi.org/10.1016/j.cct.2015.09.007> |
| Boyle, Nicola; Anderson, Jane 10.12968/bjsn.2014.9.7.339 |
| Bridge, G. L.; Willis, T. A.; Evans, C. E. L.; Roberts, K. P. J.; Rudolf, M. <https://dx.doi.org/10.1111/cch.12694> |
| Chen, C. Y.; Kao, C. C.; Hsu, H. Y.; Wang, R. H.; Hsu, S. H. <https://dx.doi.org/10.1177/1099800414565815> |
| Christison, A. L.; Evans, T. A.; Bleess, B. B.; Wang, H.; Aldag, J. C.; Binns, H. J. |
| Cordellat, A.; Padilla, B.; Grattarola, P.; Garcia-Lucerga, C.; Crehua-Gaudiza, E.; Nunez, F.; Martinez-Costa, C.; Blasco-Lafarga, C. <https://dx.doi.org/10.3390/nu12092723> |
| Cummings, C.; Crochiere, R.; Lansing, A. H.; Patel, R.; Stanger, C. <https://dx.doi.org/10.2196/32420> |
| D'Agostino, E. M.; Patel, H. H.; Hansen, E.; Mathew, M. S.; Nardi, M. I.; Messiah, S. E. <https://dx.doi.org/10.1016/j.puhe.2018.02.025> |
| Dahlin, M. S.; Reich, S. M. <https://dx.doi.org/10.1177/00178969221081805> |
| Demir Acar, M.; Bayat, M. <https://dx.doi.org/10.1089/chi.2018.0046> |
| Dinkel, D.; Tibbits, M.; Hanigan, E.; Nielsen, K.; Jorgensen, L.; Grant, K. <https://dx.doi.org/10.1080/07370016.2017.1369808> |
| Endevelt, R.; Elkayam, O.; Cohen, R.; Peled, R.; Tal-Pony, L.; Michaelis Grunwald, R.; Valinsky, L.; Porath, A.; Heymann, A. D. <https://dx.doi.org/10.3122/jabfm.2014.03.130243> |
| Erika, K. A.; Nurachmah, E.; Rustina, Y.; As'ad, S.; Nontji, W. <https://dx.doi.org/10.3923/pjn.2016.737.744> |
| Fenner, A. A.; Howie, E. K.; Davis, M. C.; Straker, L. M. <https://dx.doi.org/10.1186/s12955-016-0501-z> |
| Fenner, A. A.; Howie, E. K.; Straker, L. M.; Hagger, M. S. <https://dx.doi.org/10.1123/jsep.2015-0100> |
| Fernandez-Ruiz, V. E.; Sole-Agusti, M.; Armero-Barranco, D.; Cauli, O. <https://dx.doi.org/10.1177/1099800420987303> |
| Gehring, N. D.; Ball, G. D. C.; Perez, A.; Holt, N. L.; Neuman, D.; Spence, N.; Mercier, L.; Jetha, M. <https://dx.doi.org/10.1111/apa.14101> |
| Graziano, P. A.; Garcia, A.; Lim, C. S. <https://dx.doi.org/10.1097/DBP.0000000000000499> |
| Hadley, W.; Houck, C.; Barker, D.; Wickham, B.; Bogner, J.; Jelalian, E. <https://dx.doi.org/10.1097/DBP.0000000000000837> |
| Haghani, S.; Shahnazi, H.; Hassanzadeh, A. <https://dx.doi.org/10.5001/omj.2017.25> |
| Hao, M.; Han, W.; Yamauchi, T. <https://dx.doi.org/10.1177/1010539519848275> |
| Hayes, J. F.; Fowler, L. A.; Balantekin, K. N.; Saelens, B. E.; Stein, R. I.; Perri, M. G.; Welch, R. R.; Epstein, L. H.; Wilfley, D. E. <https://dx.doi.org/10.1002/oby.23071> |
| Heerman, W. J.; Burgess, L. E.; Escarfuller, J.; Teeters, L.; Slesur, L.; Liu, J.; Qi, A.; Samuels, L. R.; Singer-Gabella, M. 10.1016/j.cct.2018.08.006 |
| Hoeeg, Didde; Grabowski, Dan; Christensen, Ulla <https://dx.doi.org/10.1108/HE-10-2017-0056> |
| Holt, N. L.; Neely, K. C.; Newton, A. S.; Knight, C. J.; Rasquinha, A.; Ambler, K. A.; Spence, J. C.; Ball, G. D. <https://dx.doi.org/10.1016/j.jneb.2015.05.002> |
| Howie, Erin K.; McManus, Alexandra; Smith, Kyla L.; Fenner, Ashley A.; Straker, Leon M. <https://dx.doi.org/10.1089/chi.2016.0091> |
| Huang, T.; Larsen, K. T.; Jepsen, J. R. M.; Moller, N. C.; Thorsen, A. K.; Mortensen, E. L.; Andersen, L. B. <https://dx.doi.org/10.1002/oby.21209> |
| Janicke, D. M.; Lim, C. S.; Perri, M. G.; Mathews, A. E.; Bobroff, L. B.; Gurka, M. J.; Parish, A.; Brumback, B. A.; Dumont-Driscoll, M.; Silverstein, J. H. <https://dx.doi.org/10.1093/jpepsy/jsz029> |
| Jeihooni, A. K.; Heidari, M. S.; Harsini, P. A.; Azizinia, S. <https://dx.doi.org/10.1016/j.obmed.2019.100092> |
| Jelalian, E.; Darling, K.; Foster, G. D.; Runyan, T.; Cardel, M. I. <https://dx.doi.org/10.1089/chi.2022.0154> |
| Jester, A.; Kreider, K. E.; Ochberg, R.; Meek, J. <https://dx.doi.org/10.1016/j.pedhc.2017.09.006> |
| Jogova, M.; Song, J. E.; Campbell, A. C.; Warbuton, D.; Warshawski, T.; Chanoine, J. P. <https://dx.doi.org/10.1016/j.jcjd.2013.03.359> |
| Kahana, R.; Kremer, S.; Dahari, M. D.; Kodesh, E. <https://dx.doi.org/10.3390/children9010018> |
| Kahhan, N.; Hossain, M. J.; Lang, J.; Harrison, C.; Canas, J.; Wysocki, T.; Lochrie, A.; Balagopal, P. B. <https://dx.doi.org/10.1089/met.2020.0097> |
| Kanlayanee, N. I.; Tuicomepee, A.; Kiamjarasrangsi, W.; Sithisarankul, P. <https://dx.doi.org/10.3126/jnps.v37i1.16938> |
| Khumros, W.; Vorayingyong, A.; Suppapitiporn, S.; Rattananupong, T.; Lohsoonthorn, V. 10.1108/jhr-08-2018-0065 |
| Kim, H. S.; Park, J.; Park, K. Y.; Lee, M. N.; Ham, O. K. <https://dx.doi.org/10.1016/j.anr.2015.07.006> |
| Knop, C.; Singer, V.; Uysal, Y.; Schaefer, A.; Wolters, B.; Reinehr, T. <https://dx.doi.org/10.1111/j.2047-6310.2013.00212.x> |
| Kong, A. S.; Sussman, A. L.; Yahne, C.; Skipper, B. J.; Burge, M. R.; Davis, S. M. <https://dx.doi.org/10.1155/2013/575016> |
| Koo, H. C.; Poh, B. K.; Ruzita, A. T. <https://dx.doi.org/10.1186/s12889-019-7888-5> |
| Le-Jenkins, Uyen; Cartagena, Diana; Renaud, Michelle; Guston, Tina 10.1891/2380-9418.13.1.9 |
| Lek, D.; Haveman-Nies, A.; Bezem, J.; Zainalabedin, S.; Schetters-Mouwen, S.; Saat, J.; Gort, G.; Roovers, L.; van Setten, P. 10.1016/j.eclinm.2021.101217 |
| Lochrie, Amanda S.; Wysocki, Tim; Hossain, Jobayer; Milkes, Amy; Antal, Holly; Buckloh, Lisa; Canas, J.; Bobo, Elizabeth; Lang, Jason <https://dx.doi.org/10.1037/cpp0000020> |
| Looney, S. M.; Raynor, H. A. <https://dx.doi.org/10.1177/0009922814541803> |
| Lopez-Contreras, I. N.; Vilchis-Gil, J.; Klunder-Klunder, M.; Villalpando-Carrion, S.; Flores-Huerta, S. <https://dx.doi.org/10.1186/s12889-020-09339-4> |
| Mazloomy-Mahmoodabad, S. S.; Navabi, Z. S.; Ahmadi, A.; Askarishahi, M. |
| Moore, J.; Haemer, M.; Mirza, N.; Z. Weatherall Y; Han, J.; Mangarelli, C.; Hawkins, M. J.; Xanthakos, S.; Siegel, R. <https://dx.doi.org/10.3390/ijerph16101776> |
| Moore, S. M.; Borawski, E. A.; Love, T. E.; Jones, S.; Casey, T.; McAleer, S.; Thomas, C.; Adegbite-Adeniyi, C.; Uli, N. K.; Hardin, H. K.; Trapl, E. S.; Plow, M.; Stevens, J.; Truesdale, K. P.; Pratt, C. A.; Long, M.; Nevar, A. <https://dx.doi.org/10.1542/peds.2018-2185> |
| Neufeld, N. D. <https://dx.doi.org/10.1089/chi.2015.0048> |
| Nguyen, B.; Shrewsbury, V. A.; O'Connor, J.; Lau, C.; Steinbeck, K. S.; Hill, A. J.; Baur, L. A. <https://dx.doi.org/10.1093/heapro/dau110> |
| Norman, G.; Huang, J.; Davila, E. P.; Kolodziejczyk, J. K.; Carlson, J.; Covin, J. R.; Gootschalk, M.; Patrick, K. <https://dx.doi.org/10.1111/ijpo.12013> |
| Nourian, M.; Kelishadi, R.; Najimi, A. <https://dx.doi.org/10.5812/ircmj.30638> |
| Panca, M.; Christie, D.; Cole, T. J.; Costa, S.; Gregson, J.; Holt, R.; Hudson, L. D.; Kessel, A. S.; Kinra, S.; Mathiot, A.; Nazareth, I.; Wataranan, J.; Wong, I. C. K.; Viner, R. M.; Morris, S. <https://dx.doi.org/10.1136/bmjopen-2017-018640> |
| Parsons, K.; Rutkowski, E. M.; Turel, O. <https://dx.doi.org/10.1111/jspn.12235> |
| Patsopoulou, A.; Tsimtsiou, Z.; Katsioulis, A.; Malissiova, E.; Rachiotis, G.; Hadjichristodoulou, C. <https://dx.doi.org/10.1089/chi.2016.0192> |
| Pearson, E. S.; Irwin, J. D.; Burke, S. M.; Shapiro, S. |
| Pena, A.; Olson, M. L.; Ayers, S. L.; Sears, D. D.; Vega-Lopez, S.; Colburn, A. T.; Shaibi, G. Q. <https://dx.doi.org/10.3390/nu15112442> |
| Poeta, L. S.; Duarte Mde, F.; Giuliano Ide, C.; Mota, J. <https://dx.doi.org/10.1016/j.jped.2013.01.007> |
| Poeta, L. S.; Duarte, M. F. S.; Caramelli, B.; Mota, J.; Giuliano, I. C. B. <https://dx.doi.org/10.1590/S0104-42302013000100012> |
| Price, S.; Ferisin, S.; Sharifi, M.; Steinberg, D.; Bennett, G.; Wolin, K. Y.; Horan, C.; Koziol, R.; Marshall, R.; Taveras, E. M. <https://dx.doi.org/10.1080/10810730.2015.1018582> |
| Quattrin, T.; Cao, Y.; Paluch, R. A.; Roemmich, J. N.; Ecker, M. A.; Epstein, L. H. <https://dx.doi.org/10.1542/peds.2016-2755> |
| Rabiei, L.; Heydarabadi, A. B.; Tavassoli, E.; Abbasi, M.; Khayeri, F.; Masoudi, R. 10.22038/ijp.2017.24653.2080 |
| Rauber, S. B.; Castro, H. O.; Marinho, A.; Vicente, J. B.; Ribeiro, H. L.; Monteiro, L. Z.; Praca, I. R.; Simoes, H. G.; Campbell, C. S. G. <https://dx.doi.org/10.1177/0260106018771519> |
| Reilly, K. C.; Briatico, D.; Irwin, J. D.; Tucker, P.; Pearson, E. S.; Burke, S. M. <https://dx.doi.org/10.3390/ijerph16122171> |
| Rieder, J.; Khan, U. I.; Heo, M.; Mossavar-Rahmani, Y.; Blank, A. E.; Strauss, T.; Viswanathan, N.; Wylie-Rosett, J. <https://dx.doi.org/10.1089/chi.2012.0147> |
| Rito, A. I.; Carvalho, M. A.; Ramos, C.; Breda, J. <https://dx.doi.org/10.1017/S1368980013000244> |
| Rune, K. T.; Mulgrew, K.; Sharman, R.; Lovell, G. P. <https://dx.doi.org/10.1071/HE14060> |
| Sallinen, B. J.; Schaffer, S.; Woolford, S. J. <https://dx.doi.org/10.1089/chi.2012.0106> |
| Savoye, M.; Caprio, S.; Dziura, J.; Camp, A.; Germain, G.; Summers, C.; Li, F.; Shaw, M.; Nowicka, P.; Kursawe, R.; DePourcq, F.; Kim, G.; Tamborlane, W. V. <https://dx.doi.org/10.2337/dc13-1571> |
| Sen, M.; Uzuner, A.; Akman, M.; Bahadir, A. T.; Borekci, N. O.; Viggiano, E. <https://dx.doi.org/10.1007/s00431-018-3177-z> |
| Shanthi, M.; Kanniammal, C.; Mahendra, J.; Valli, G. <https://dx.doi.org/10.5958/0976-5506.2019.00052.4> |
| Sherwood, N. E.; Levy, R. L.; Seburg, E. M.; Crain, A. L.; Langer, S. L.; JaKa, M. M.; Kunin-Batson, A.; Jeffery, R. W. <https://dx.doi.org/10.1111/ijpo.12523> |
| Soltero, E. G.; Olson, M. L.; Williams, A. N.; Konopken, Y. P.; Castro, F. G.; Arcoleo, K. J.; Keller, C. S.; Patrick, D. L.; Ayers, S. L.; Barraza, E.; Shaibi, G. Q. <https://dx.doi.org/10.1002/oby.22300> |
| Stovitz, S. D.; Berge, J. M.; Wetzsteon, R. J.; Sherwood, N. E.; Hannan, P. J.; Himes, J. H. <https://dx.doi.org/10.1089/chi.2013.0107> |
| Sweat, V.; Bruzzese, J. M.; Fierman, A.; Mangone, A.; Siegel, C.; Laska, E.; Convit, A. <https://dx.doi.org/10.1007/s10900-015-0041-1> |
| Tabak, R. G.; Dsouza, N.; Schwarz, C. D.; Quinn, K.; Kristen, P.; Haire-Joshu, D. <https://dx.doi.org/10.1186/s12889-018-5466-x> |
| Taveras, E. M.; Marshall, R.; Sharifi, M.; Avalon, E.; Fiechtner, L.; Horan, C.; Gerber, M. W.; Orav, E. J.; Price, S. N.; Sequist, T.; Slater, D. <https://dx.doi.org/10.1001/jamapediatrics.2017.1325> |
| Taveras, E. M.; Marshall, R.; Sharifi, M.; Avalon, E.; Fiechtner, L.; Horan, C.; Orav, J.; Price, S. N.; Sequist, T.; Slater, D. <https://dx.doi.org/10.1016/j.cct.2015.09.022> |
| Thoren, Annelie; Janson, Annika; Persson, Margareta <https://dx.doi.org/10.1111/apa.15798> |
| Top, Fadime Üstüner; Kaya, Barış; Tepe, Banu; Avci, Esin 10.1007/s10597-019-00415-7 |
| Tripicchio, G. L.; Ammerman, A. S.; Neshteruk, C.; Faith, M. S.; Dean, K.; Befort, C.; Ward, D. S.; Truesdale, K. P.; Burger, K. S.; Davis, A. <https://dx.doi.org/10.1089/chi.2017.0021> |
| Tucker, J. M.; DeFrang, R.; Orth, J.; Wakefield, S.; Howard, K. <https://dx.doi.org/10.3390/nu11030498> |
| Tucker, J. M.; Howard, K.; DeLaFuente, K.; Cadieux, A.; Yee, K. E. <https://dx.doi.org/10.1111/cob.12215> |
| van den Eynde, E.; Camfferman, R.; Putten, L. R.; Renders, C. M.; Seidell, J. C.; Halberstadt, J. <https://dx.doi.org/10.1089/chi.2020.0070> |
| van der Baan-Slootweg, O.; Benninga, M. A.; Beelen, A.; van der Palen, J.; Tamminga-Smeulders, C.; Tijssen, J. G.; van Aalderen, W. M. <https://dx.doi.org/10.1001/jamapediatrics.2014.521> |
| van Middelkoop, M.; Ligthart, K. A. M.; Paulis, W. D.; van Teeffelen, J.; Kornelisse, K.; Koes, B. W. <https://dx.doi.org/10.1093/fampra/cmx056> |
| Verbeken, S.; Braet, C.; Naets, T.; Houben, K.; Boendermaker, W. <https://dx.doi.org/10.1016/j.appet.2017.12.029> |
| Verrotti, A.; Agostinelli, S.; D'Egidio, C.; Di Fonzo, A.; Carotenuto, M.; Parisi, P.; Esposito, M.; Tozzi, E.; Belcastro, V.; Mohn, A.; Battistella, P. A. <https://dx.doi.org/10.1111/j.1468-1331.2012.03771.x> |
| Vlaev, I.; Taylor, M. J.; Taylor, D.; Gately, P.; Gunn, L. H.; Abeles, A.; Kerkadi, A.; Lothian, J.; Jreige, S. K.; Alsaadi, A.; Al-Kuwari, M. G.; Ghuloum, S.; Al-Kuwari, H.; Darzi, A.; Ahmedna, M. <https://dx.doi.org/10.1186/s12889-021-10838-1> |
| Watson, Libby A.; Baker, Martyn C.; Chadwick, Paul M. <https://dx.doi.org/10.1111/bjhp.12175> |
| Wilson, D. K.; Sweeney, A. M.; Quattlebaum, M.; Loncar, H.; Kipp, C.; Brown, A. <https://dx.doi.org/10.3390/nu13061745> |
| Wilson, D. K.; Sweeney, A. M.; Van Horn, M. L.; Kitzman, H.; Law, L. H.; Loncar, H.; Kipp, C.; Brown, A.; Quattlebaum, M.; McDaniel, T.; St George, S. M.; Prinz, R.; Resnicow, K. <https://dx.doi.org/10.1093/abm/kaab110> |
| Windham, M. E.; Hastings, E. S.; Anding, R.; Hergenroeder, A. C.; Escobar-Chaves, S. L.; Wiemann, C. M. <https://dx.doi.org/10.1016/j.jand.2014.04.014> |
| Wongtongtair, S.; Iamsupasit, S.; Somrongthong, R.; Kumar, R.; Yamarat, K. <https://dx.doi.org/10.12688/f1000research.51156.2> |
| Yeh, Y.; Hartlieb, K. B.; Danford, C.; Catherine Jen, K. L. <https://dx.doi.org/10.1007/s40615-017-0399-0> |
| Yu, Hong-jie; Li, Fang; Hu, Yong-feng; Li, Chang-feng; Yuan, Shuai; Song, Yong; Zheng, Miaobing; Gong, Jie; He, Qi-qiang. https://dx.doi.org/10.3390/nu12010194 |

**Wrong publication n=31**

| **Authors; DOI** |
| --- |
| Barnett, Andrietta Wright |
| Brito, F. A.; Zoellner, J. M.; Hill, J.; You, W.; Alexander, R.; Hou, X.; Estabrooks, P. A. https://dx.doi.org/10.1177/2158244019837313 |
| Durrer-Schutz, D.; Kowatsch, T.; L'Allemand-Jander, D.; Buchter, D.; Schutz, Y. https://dx.doi.org/10.1111/%28ISSN%291467-789X |
| Fals, A. M. https://dx.doi.org/10.1542/peds.144.2-MeetingAbstract.221 |
| Gago, C.; Beckerman-Hsu, J.; Figueroa, R.; Aftosmes-Tobio, A.; Lansburg, K.; Garcia, E.; Lefebvre, L.; Davison, K. https://dx.doi.org/10.1002/oby.23063 |
| Gottschalk, L. B.; Stevens, D. L.; Jensen, A.; Beyrouty, M.; Skursky, N.; Murphy, J.; Schwartz, M.; Sherman, S. https://dx.doi.org/10.1016/j.jadohealth.2014.10.209 |
| Gutierrez, W.; Tung, A.; Sihotang, C.; Westerberg, M.; Baum, M. https://dx.doi.org/10.231/JIM.0b013e31827d3ac9 |
| Holmberg, Christopher; Berg, Christina; Dahlgren, Jovanna; Lissner, Lauren; Chaplin, John Eric 10.1177/1460458218759699 |
| Israt, S.; Roy, S. K.; Ferdouse, K.; Jahan, K. https://dx.doi.org/10.1159/000354245 |
| Kebbe, M.; Perez, A.; Buchholz, A.; McHugh, T. F.; Scott, S. S.; Richard, C.; Mohipp, C.; Dyson, M. P.; Ball, G. D. C. https://dx.doi.org/10.1371/journal.pone.0209219 |
| Kebbe, Maryam; Perez, Arnaldo; Buchholz, Annick; McHugh, Tara-Leigh F.; Scott, Shannon D.; Richard, Caroline; Dyson, Michele P.; Ball, Geoff D. C. 10.1080/17538068.2020.1765126 |
| Martin, C.; Apolzan, J.; Hawkins, K.; Hall, L.; Davis, A.; Chatham, J. https://dx.doi.org/10.1159/000468958 |
| Randhawa, S.; Sorrells, R.; Engebretsen, S.; Yi-Frazier, J. P.; Early, K. B. |
| Rauber, S. B.; Ribeiro, H. L.; Marinho, A.; Madrid, B.; Vicente, J. B.; Campbell, C. S. G. 10.1249/01.mss.0000519402.89527.dd |
| Resnicow, Ken; Sonneville, Kendrin R.; Naar, Sylvie 10.1542/peds.2018-2471 |
| Ribeiro, H. L.; Rauber, S. B.; Campbell, C. S. 10.1249/01.mss.0000562093.34951.a7 |
| Riiser, Kirsti; Londal, Knut; Ommundsen, Yngvar; Misvaer, Nina; Helseth, Solvi https://dx.doi.org/10.1177/0969733014524761 |
| Robinson, T. N.; Matheson, D.; Desai, M.; Wilson, D. M.; Weintraub, D. L.; Haskell, W. L.; McClain, A.; McClure, S.; Banda, J. A.; Sanders, L. M.; Haydel, K. F.; Killen, J. D. https://dx.doi.org/10.1016/j.cct.2013.09.001 |
| Rosenkranz, Richard R.; Cull, Brooke J.; Rosenkranz, Sara K.; Dzewaltowski, David A. 10.1001/jamanetworkopen.2022.16720 |
| Sharifi, Mona; Dryden, Eileen M.; Horan, Christine M.; Price, Sarah; Marshall, Richard; Hacker, Karen; Finkelstein, Jonathan A.; Taveras, Elsie M. https://dx.doi.org/10.2196/jmir.2780 |
| Stasinaki, A.; Brogle, B.; Buchter, D.; Shih, C. H. I.; Heldt, K.; White, C.; Ruegger, D.; Filler, A.; Gindrat, P.; Durrer, D.; Farpour-Lambert, N.; Kowatsch, T.; Lallemand, D. |
| Taveras, E. M.; Marshall, R.; Sharifi, M.; Avalon, E.; Fiechtner, L.; Horan, C.; Gerber, M.; Orav, J.; Price, S. N.; Sequist, T.; Slater, D. https://dx.doi.org/10.25302/3.2018.IH.13046739 |
| Tompkins, C. L.; Laurent, J.; Brock, D. W. https://dx.doi.org/10.1089/chi.2017.0003 |
| Tripicchio, Gina Lauren |
| Watts, A. W.; Lovato, C. Y.; Barr, S. I.; Hanning, R. M.; Masse, L. C. https://dx.doi.org/10.1017/S1368980015000786 |
| Weekley, A.; Calkin, K.; Buffalo, J.; Boekel, S.; Mullis, R.; Anderson, A. |
| Werk, L. N.; Hossain, J.; Martinez, A.; Abatemarco, A.; Carlo, V.; Barnini, N.; McCahan, S.; Pennington, C.; Phan, T. L.; Bunnell, T.; Hassink, S. G. https://dx.doi.org/10.1542/peds.144.2-MeetingAbstract.219 |
| Yudkin, J.; Allicock, M.; Barlow, S. https://dx.doi.org/10.1002/oby.23626 |
| Zoellner, J. M.; You, W.; Hill, J. L.; Brock, D. P.; Yuhas, M.; Alexander, R. C.; Price, B.; Estabrooks, P. A. https://dx.doi.org/10.1016/j.cct.2019.06.015 |
| Zoellner, J.; Hill, J. L.; You, W.; Brock, D.; Frisard, M.; Alexander, R.; Brito, F.; Price, B.; Marshall, R.; Estabrooks, P. A. |

**Wrong population n=9**

| **Authors; DOI** |
| --- |
| Brown, C. W.; Alexander, D. S.; Warren, C. A.; Anderson-Booker, M. https://dx.doi.org/10.1007/s40615-016-0257-5 |
| Brown, E. C.; Buchan, D. S.; Drignei, D.; Wyatt, F. B.; Kilgore, L.; Cavana, J.; Baker, J. S. https://dx.doi.org/10.3389/fped.2018.00137 |
| Diao, H.; Wang, H.; Yang, L.; Li, T. https://dx.doi.org/10.1186/s12955-020-01459-0 |
| Koch, Pamela Ann; Contento, Isobel R.; Gray, Heewon L.; Burgermaster, Marissa; Bandelli, Lorraine; Abrams, Emily; Di Noia, Jennifer https://dx.doi.org/10.1016/j.jneb.2018.12.001 |
| Larsen, L. M.; Hertel, N. T.; Molgaard, C.; Christensen, R. D.; Husby, S.; Jarbol, D. E. https://dx.doi.org/10.3109/02813432.2015.1067511 |
| Maatoug, J.; Fredj, S. B.; Msakni, Z.; Dendana, E.; Sahli, J.; Harrabi, I.; Chouikha, F.; Boughamoura, L.; Slama, S.; Farpour-Lambert, N.; Ghannem, H. https://dx.doi.org/10.1515/ijamh-2015-0035 |
| Nezondet, C.; Gandrieau, J.; Bourrelier, J.; Nguyen, P.; Zunquin, G. https://dx.doi.org/10.3390/children10060956 |
| Shomaker, L. B.; Tanofsky-Kraff, M.; Matherne, C. E.; Mehari, R. D.; Olsen, C. H.; Marwitz, S. E.; Bakalar, J. L.; Ranzenhofer, L. M.; Kelly, N. R.; Schvey, N. A.; Burke, N. L.; Cassidy, O.; Brady, S. M.; Dietz, L. J.; Wilfley, D. E.; Yanovski, S. Z.; Yanovski, J. A. https://dx.doi.org/10.1002/eat.22741 |
| Singh, P. N.; Steinbach, J.; Nelson, A.; Shih, W.; D'Avila, M.; Castilla, S.; Jordan, M.; McCarthy, W. J.; Hayes-Bautista, D.; Flores, H. https://dx.doi.org/10.3390/ijerph17134849 |

**Wrong outcomes n=1**

| **Authors; DOI** |
| --- |
| Te'o, D. T.; Wild, C. E. K.; Willing, E. J.; Wynter, L. E.; O'Sullivan, N. A.; Hofman, P. L.; Maessen, S. E.; Derraik, J. G. B.; Anderson, Y. C. https://dx.doi.org/10.3390/nu14204363 |
